# Multidecadal variability in Atlas cedar growth in Northwest Africa during the last 850 years: Implications for dieback and conservation of an endangered species

**DOI:** 10.1016/j.dendro.2019.05.003

**Published:** 2019-08

**Authors:** Kelsey Copes-Gerbitz, William Fletcher, Jonathan G.A. Lageard, Mustapha Rhanem, Sandy P. Harrison

**Affiliations:** a Department of Geography, School of Environment, Education and Development, University of Manchester, Manchester, M13 9PL, UK; c Division of Geography & Environmental Management, School of Science and the Environment, Manchester Metropolitan University, Manchester, M1 5GD, UK; d Unité de Botanique et Écologie Montagnarde, Université Moulay Ismail, Faculté des Sciences, BP 11201, Zitoune, Meknès, Morocco; e School of Archaeology, Geography and Environmental Science (SAGES), University of Reading, Reading, RG6 6AB, UK

**Keywords:** *Cedrus atlantica*, Atlas cedar, Forest dieback, Dendrochronology, Multidecadal, Variability, Conservation

## Abstract

Widespread forest dieback is a phenomenon of global concern that requires an improved understanding of the relationship between tree growth and climate to support conservation efforts. One priority for conservation is the Atlas cedar (*Cedrus atlantica*), an endangered species exhibiting dieback throughout its North African range. In this study, we evaluate the long-term context for recent dieback and develop a projection of future *C. atlantica* growth by exploring the periodic variability of its growth through time. First, we present a new *C. atlantica* tree-ring chronology (1150–2013 CE) from the Middle Atlas mountains, Morocco. We then compare the new chronology to existing *C. atlantica* chronologies in Morocco and use principal components analysis (PCA) to isolate the common periodic signal from the seven longest available records (PCA_7_, 1271–1984 CE) in the Middle and High Atlas portions of the *C. atlantica* range. PCA_7_ captures 55.7% of the variance and contains significant multidecadal (˜95 yr, ˜57 yr, ˜21 yr) periodic components, revealed through spectral and wavelet analyses. Parallel analyses of historical climate data (1901–2016 CE) suggests that the multidecadal growth signal originates primarily in growing season (spring and summer) precipitation variability, compounded by slow-changing components of summer and winter temperatures. Finally, we model the long-term growth patterns between 1271–1984 CE using a small number (three to four) of harmonic components, illustrating that suppressed growth since the 1970s – a factor implicated in the dieback of this species – is consistent with recurrent climatically-driven growth declines. Forward projection of this model suggests two climatically-favourable periods for growth in the 21st century that may enhance current conservation actions for the long-term survival of the *C. atlantica* in the Middle and High Atlas mountains.

## 1 Introduction

Quantifying the impact of climate change on ecological systems is one of the greatest scientific challenges of today (Easterling et al., 2000; Walther et al., 2002). Worldwide, climate change is already linked to widespread forest dieback in a variety of ecosystems (Allen et al., 2010; Settele et al., 2014; McDowell and Allen, 2015; Neumann et al., 2017; Boulton et al., 2017). In northwest Africa, where drought is expected to intensify (Knippertz et al., 2003; Born et al., 2008), there is high to very high confidence that forested ecosystems are vulnerable to a biome shift under varying climate change scenarios (Gonzalez et al., 2010). A primary component of northwest African forests is the culturally-iconic, long-lived Atlas cedar (*Cedrus atlantica* (Endl.) Manetti ex Carrière; hereafter referred to as *C. atlantica*), listed as an endangered species in 2013 (M’hirit and Benzyane, 2006; Thomas, 2013). The current range of *C. atlantica* is in mountainous regions (1500–2,600 m above sea level) of Morocco and Algeria, with the core of the population in the Middle Atlas mountains; fossil data indicates this Middle Atlas population is relatively young (˜10kya) (Benabid, 1994; Terrab et al., 2008). Existing populations of *C. atlantica* are severely fragmented (Thomas, 2013), with a 75% decrease in range since the 1940s due to anthropogenic pressure and climatic changes (Quézel, 2000; Départment des Forêts, 2010). Although ongoing mass mortality is linked to the onset of a severe drought in the 1970s, drought-induced dieback events are not solely a modern phenomenon; previous research indicates a dieback event in 1872–1882 Common Era, CE (Bentouati, 2008; Linares et al., 2013; Kherchouche et al., 2012; Touchan et al., 2008).

Understanding the long-term context of climate impacts on tree growth places recent dieback in perspective and provides important insights for forest resilience (Gazol et al., 2018). Tree-ring records can be used to investigate interannual to multidecadal and centennial climate trends because of the sensitivity of tree growth to climate in arid and semi-arid regions (e.g. Fritts, 1976; St. George, 2014). A large number of dendroclimatological studies have used the drought-sensitive *C. atlantica* to reconstruct climate variability across multiple spatial scales, including: local temperature and precipitation (*e.g.*, Till, 1987; Berger et al., 1979; Till and Guiot, 1990; Ilmen et al., 2013); regional droughts (*e.g.*, Chbouki et al., 1995; Esper et al., 2007; Touchan et al., 2011); and dominant modes of climate variability (*e.g.*, Glueck and Stockton, 2001; Trouet et al., 2009; Wassenberg et al., 2013). Several studies highlight a complex response of tree growth to both temperature and precipitation (*e.g.*, Chbouki et al., 1995; Esper et al., 2007) and identify the variability of this relationship through time (*e.g.*, Dutilleul and Till, 1992; Chbouki et al., 1995). While these studies effectively characterise climate-growth relationships of *C. atlantica* to reconstruct past climate variability, there is a lack of understanding of the multidecadal periodic dynamics driving *C. atlantica* growth. Given the projected intensification of drought in northwest Africa (Meehl and Tebaldi, 2004; Schilling et al., 2012), understanding of the impacts of multidecadal climate variability on *C. atlantica* growth will provide important historical context and future insights for the survival of this endangered species and associated montane forest type.

In this study, we evaluate the current *C. atlantica* dieback in the context of growth patterns during the last 850 years to understand whether dieback episodes are consistent across the range of *C. atlantica* and have their origin in multidecadal climate variability. To do so, we develop a new tree-ring chronology from the most sensitive old-growth forests in the Middle Atlas and integrate this chronology with existing *C. atlantica* tree-ring records to derive a new regional growth signal covering the period 1271–1984 CE. Our objectives are to (i) explore variability in *C. atlantica* chronologies across its range; (ii) test for the presence of significant periodic components in a regional growth signal; (iii) compare the regional growth signal to regional temperature and precipitation; and (iv) model the potential future growth of *C. atlantica*. Through this approach, we characterise the multidecadal growth signals in *C. atlantica* chronologies and consider the importance of a long-term perspective in addressing the forest dieback issue.

## 2 Materials and methods

### 2.1 Study area

**Fig. 1:**
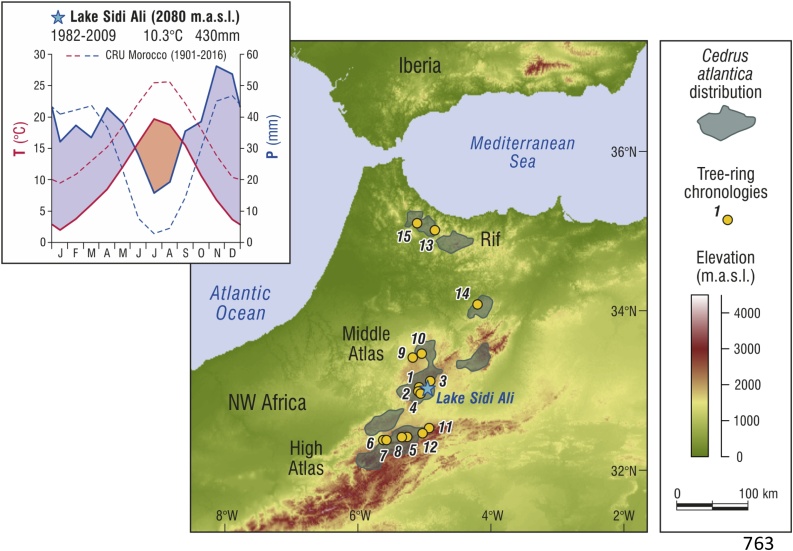
Location of the Lake Sidi Ali study site in northwest Africa and other *Cedrus atlantica* chronologies from the ITRDB (see Table 1 for data sources). Inset box shows climate data for the Lake Sidi Ali station (solid lines), courtesy of the Agence du Bassin Hydraulique du Sebou, with summary of CRU historical climatology for all Morocco (dotted lines, Harris et al., 2014). Shaded areas show the Moroccan distribution of *Cedrus atlantica*, following Linares et al. (2011). GTOPO30 digital elevation data from the U.S. Geological Survey. (For interpretation of the references to colour in this figure legend, the reader is referred to the web version of this article).

The core of the *C. atlantica* population is in the Middle (Moyen) Atlas Mountains of Morocco, within a range that includes the High and Rif Atlas mountains of Morocco and the northern mountains in Algeria (Fig. 1; Pujos, 1966; Quézel, 2000; Thomas, 2013). The *C. atlantica* forest sampled in this study is located near Lake Sidi Ali (33° 03′ N, 05° 00′ W, 2080 m.a.s.l.), one of the largest lakes in Morocco situated at the southern margins of the Middle Atlas in a previously unstudied area. Pollen analyses of the Lake Sidi Ali sediments (Zielhofer et al., 2017; Campbell et al., 2017) show that a local population of *C. atlantica* has been present since at least 6.3 kya (thousand years ago).

The climate of the study area is dominated by sub-tropical high pressure in summer and Atlantic westerly circulation in winter. Station data from Lake Sidi Ali indicates average monthly temperature ranges from 34 °C in July (mean maxima) to −10 °C in January (mean minima) (Fig. 1, inset). Average yearly precipitation for 1982–2009 CE was 430 mm, with maxima in November-December and April, but with high interannual variability (s.d. 130 mm; see also Sayad et al., 2011). The drier summers are marked by short-lived, heavy rain events, while winter precipitation is more constant and often falls as snow. Compared with long-term (1901–2016) mean conditions for Morocco, the climate at Lake Sidi Ali is cooler and displays a shorter drought season within the prevailing Mediterranean climate pattern of humid winters and summer drought (Fig. 1, inset).

The montane forest vegetation cover, degraded by grazing and wood harvesting, corresponds to the high-altitude, semi-arid *Cedrus* series (Achhal et al., 1980). *C. atlantica* is dominant, interspersed with Spanish juniper (*Juniperus thurifera* Lam.) and patches of multi-stemmed holm oak (*Quercus ilex* subsp. *ballota* (Desf.) Samp.). The *C. atlantica* in the Sidi Ali catchment have two different growth forms: (i) large, relatively undisturbed, open grown trees with intact lower branches and (ii) smaller trees without lower branches. The larger trees show evidence of dieback, including a yellowing of needles, visible rot, defoliated branches and dead tops (Rhanem, 2011).

### 2.2 Chronology development

The sampled stand of *C. atlantica* on the southeastern margins of Lake Sidi Ali was chosen because its northerly exposure, limestone substrate and elevation make it particularly sensitive to climate stress (Till, 1987). The sampled stand is fenced off as part of a regional restoration effort for this endangered species; permission for coring was received from the Haut Commissariat aux Eaux et Forêts et à la Lutte Contre la Désertification (High Commission for Water and Forests and the Fight Against Desertification). We only sampled open-grown trees with lower branches to minimise possible impacts of inter-tree competition and wood harvesting; both live and dead trees of varying diameter at breast height (43.2–184.5 cm) were sampled to minimise potential effects of age or health on the desired climate signal. Cores from 33 trees were extracted perpendicular to the slope using increment borers as low to the root collar as possible while avoiding buttressed roots and visible scars. Since *C. atlantica* is an endangered species and many trees show signs of poor health, only one core per tree was taken to minimise potential damage.

A single growth chronology was constructed from the individual tree-ring series using the standard dendrochronological approach (Stokes and Smiley, 1946). Cores were mounted, sanded and scanned at high resolution to measure ring widths using CooRecorder (Larsson, 2011a). Samples were cross-dated in CDendro (Larsson, 2011b) against a regional master *C. atlantica* chronology that included 114 individual series from 71 trees taken from two tree-ring data sets (morc018 and morc023; Esper et al., 2007) near Lake Sidi Ali available through the International Tree-Ring Data Bank (ITRDB). Internal cross-dating of the Lake Sidi Ali chronology was statistically verified using COFECHA (Holmes, 1983); series with an internal correlation ≥0.480 (at p < 0.001) were retained for additional analyses (n = 29). After multiple iterations using different detrending techniques in CRUST (Melvin and Briffa, 2014), signal-free detrending of the tree-ring series was made by fitting a 300-year spline to the individual series and subsequent standardization to generate the Lake Sidi Ali tree ring index (TRI) using the standard chronology output (Speer, 2010). This spline-fitting technique was selected to retain maximum variance at multi-decadal time scales (Speer, 2010). The resulting chronology met the recommended quality characteristics required to undertake further climate-growth analyses (Fritts, 1976; Speer, 2010).

### 2.3 Identification of regional growth signal

All available tree-level records (n = 15) for *C. atlantica* were obtained from the ITRDB; only tree-level measurements were selected to ensure different detrending techniques between chronologies did not affect the results. Each series was detrended in CRUST (Melvin and Briffa, 2014) using the same 300-yr spline as described above to create comparable individual site-level chronologies. The resulting standard chronologies were used as the input to a correlation matrix to evaluate between-site correlations and assess whether the Lake Sidi Ali record is regionally representative. Principal components analysis (PCA) was then used to extract the dominant common signal from the individual site-level *C. atlantica* chronologies. PCA was performed on two different subsets of the chronologies: (i) a set with a multi-centennial record (1271–1984 CE; n = 7, hereafter referred to as PCA_7_) and (ii) a full set of all available chronologies covering a shorter timespan (1845–1984 CE; n = 16, hereafter referred to as PCA_16_). The choice of seven chronologies for PCA_7_ reflects a trade-off between length and number of the compiled chronologies; increasing the number to eight chronologies reduces the length of the chronology by 221 years, while reducing the number to six chronologies only extends the length by 18 years. PCA was undertaken on the covariance matrix and the number of significant components was evaluated against a “broken-stick” model (King and Jackson, 1999). Both inter-site correlations, site loadings on the first principal components and correlations between the site chronologies and the first principal components were evaluated to assist in the interpretation of the principal components.

### 2.4 Timeseries analyses

Timeseries analyses were performed to identify periodic components and their temporal expression in the common regional growth signal for long *C. atlantica* chronologies (first principal component of the multi-centennial record, PCA_7_). Spectral analysis was performed using RedFit (Schulz and Mudelsee, 2002), whereby the frequency content of the timeseries is evaluated against a first order autoregressive (AR1) red noise model. RedFit analysis was implemented in the PAST software package (Hammer, 2014) selecting the “Welch” window, three overlapping segments and an oversampling factor of four. The upper confidence interval of the AR1 noise can be calculated based on a χ^2^ distribution for specific significance levels to allow detection of statistically significant peaks (Schulz and Mudelsee, 2002). The stringent critical false-alarm level was used ([1–1/n] × 100%, where n = number of data points within each overlapping segment), which is recommended for exploratory analysis of timeseries where the possibility of false positives is high (Thomson, 1990). Second, wavelet analysis of PCA_7_ was undertaken using the continuous wavelet transform (Torrence and Compo, 1998) to detect periodic components that are confined to certain time intervals or which change in frequency over time and might not be revealed by traditional spectral approaches (Cazelles et al., 2008). Prior to wavelet analysis, the frequency distribution of the timeseries was checked to confirm that it approaches a normal distribution (Grinsted et al., 2004). Then, the wavelet transform was computed and the wavelet power spectrum was plotted for each timeseries by applying the Morlet wavelet which offers good frequency localization (Cazelles et al., 2008), with a temporal resolution of 1 yr and a selected range of Fourier periods for wavelet decomposition of 2 yr to 512 yr. Zero padding of the time-series is required to avoid false periodic detection but can lead to edge effects; the area where these may be significant is identified by the “cone of influence”, which is plotted on the wavelet diagrams. Wavelet analysis was implemented in the R package WaveletComp v1.0 (Rösch and Schmidbauer, 2016). Statistical significance was assessed using 1000 Monte Carlo simulations and results significant at the 95% confidence level (p < 0.05) are reported. Third, we model significant periodicities in PCA_7_ using sinusoidal regression and forward selection of best-fit sinusoids. The selection procedure was implemented in PAST, based on a least-squares criterion and singular value decomposition (Press et al., 1992). We report the percentage of variance explained (coefficient of determination (R^2^) × 100) as a measure of the goodness of fit models based on the sum of one to four individual regression terms, as well as composite models for three and four terms, respectively; all models are significant at the 95% confidence level at least. The regression modelling allows for projection of the modelled components forward in time; modelled values for PCA_7_ were then projected for the interval 1985–2100 CE to show the anticipated possible future growth trends corresponding to the different significant periodic components of the historical tree-ring records.

### 2.5 Regional climatology

Due to the short length and frequent missing values in the local Lake Sidi Ali climate station data, a traditional calibration and reconstruction exercise was not possible at the site level. Instead, we evaluate the new Lake Sidi Ali tree-ring chronology dataset as well as the integrated PCA_7_ signal against annual and seasonal temperature and precipitation data from a regional climatology product, the CRU CY v. 4.02 Morocco climatology (Harris et al., 2014) which covers 1901 to 2016 CE. Previous studies demonstrate that a growing season (Feb-Oct) drought index (scanning Palmer Drought Severity Index) that integrates temperature and precipitation influence on tree growth has high explanatory power for *C. atlantica* variability in Morocco (Esper et al., 2007). This study retains the use of separate temperature and precipitation datasets to permit the independent evaluation of multidecadal components of historical temperature and precipitation. Correlation analysis for PCA_7_ and annual and seasonal climatology (1901–1984 CE) was used to identify the likely drivers and main seasons of climate influence. Results are considered significant at the 95% confidence level (p < 0.05); no Bonferroni correction was applied to avoid the risk of Type II errors (Perneger, 1998). Best-fit sinusoidal regression was applied to the historical climate data (1901–2016 CE) to detect and illustrate multidecadal components.

## 3 Results

### 3.1 Lake Sidi Ali chronology

**Fig. 2:**
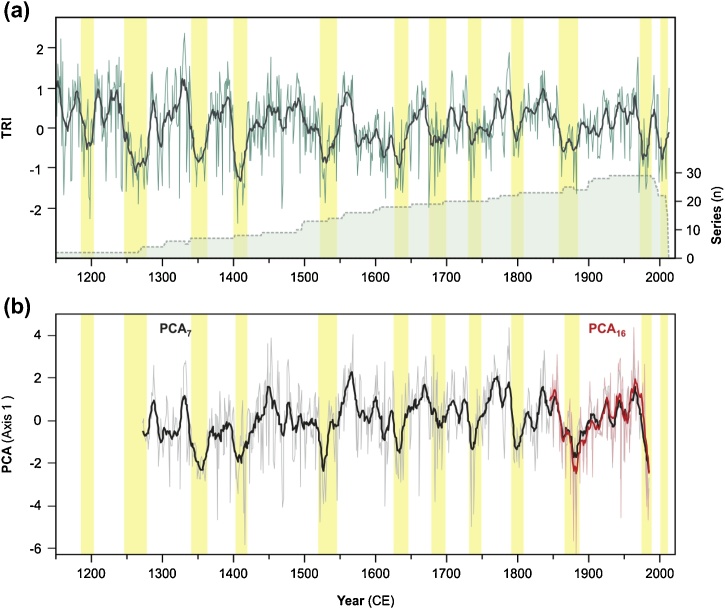
Dendrochronological results from the Lake Sidi Ali catchment and integration with *C. atlantica* chronologies from the International Tree-Ring Data Bank (n = 15; see Table 1), showing (a) the detrended and standardized Lake Sidi Ali tree ring index (TRI) with 11 yr moving average and the series depth (green shaded curve) and (b) scores on the first principal component for PCA of seven sites with long chronologies from 1271 CE (grey and black lines showing annual and 11 yr moving average, respectively) and PCA of sixteen sites with chronologies from 1845 CE (pink and red lines showing annual and 11 yr moving average, respectively). Vertical yellow bars in (a) show multidecadal low growth intervals in the Sidi Ali TRI; these bars are replicated in (b) to highlight strong similarities with negative scores on the first principal components of PCA_7_ and PCA_16_. (For interpretation of the references to colour in this figure legend, the reader is referred to the web version of this article).

The Lake Sidi Ali chronology (TRI) is based on 29 trees or series (Fig. 2a; Supplementary Table 1); series from four trees were not included in the chronology because of unclear ring patterns and a low correlation (<0.484) with the regional master chronology. The length of the Lake Sidi Ali TRI is 864 years (1150–2013 CE), with a mean series length of 409.5 years. The series intercorrelation is 0.665 while the average mean sensitivity is 0.366, both within recommended ranges for exploring climate-growth relationships (Fritts, 1976; Speer, 2010). The post-1282 CE component of the TRI exceeds the minimum expressed population signal (EPS) of 0.85 required to ensure common stand-level variability between individuals. The minimum EPS for the interval 1150 to 1282 CE exceeds 0.67. The Lake Sidi Ali TRI displays strong variability on multidecadal timescales and recurrent periods of low growth (yellow shading, Fig. 2a), including recent low-growth intervals in the 1970s and early 2000s; this early 2000s low-growth interval is not captured by other *C. atlantica* data sets because of their time period covered.

### 3.2 Regional growth signal

**Fig. 3:**
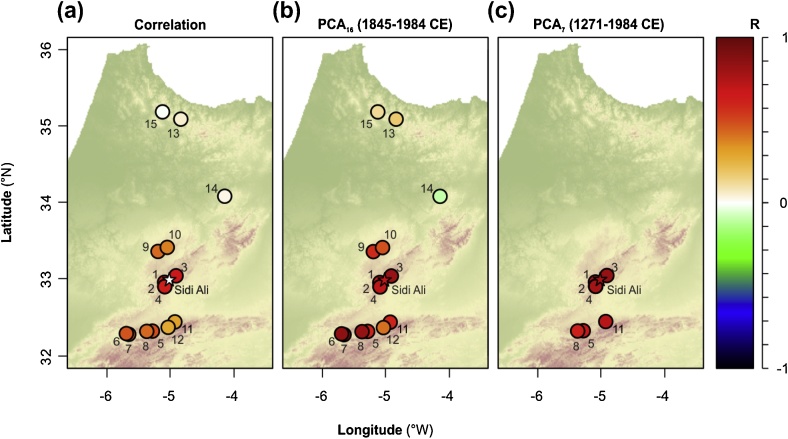
Relationships between Lake Sidi Ali *C. atlantica* chronology and other *C. atlantica* records showing (a) correlation coefficients between the Sidi Ali chronology (star) and the other *C. atlantica* chronologies; correlation coefficients between the chronologies and the first principal component of (b) PCA_16_ and (c) PCA_7_. (For interpretation of the references to colour in this figure legend, the reader is referred to the web version of this article).

The Sidi Ali TRI is significantly (p < 0.01) correlated to chronologies in the Middle (R = 0.38 to 0.70) and High (R = 0.28 to 0.49) Atlas, but not significantly correlated to chronologies in the Rif Atlas (Table 1; Fig. 3a). Correlation coefficients against the Rif Atlas chronologies were all <0.06. The four southern Middle Atlas chronologies with correlations > 0.60 were those that were within 15 km of the Lake Sidi Ali site, highlighting that the Lake Sidi Ali chronology reinforces a coherent growth signal from this portion of the *C. atlantica* range.

**Table 1:** Moroccan *C. atlantica* tree-level chronologies available on the International Tree-Ring Data Bank.

Number	Name	Elevation (m.a.s.l)	Citation	Period of record (CE)	Correlation against Sidi Ali TRI^*^	PCA_16_ Axis 1 Loading	PCA_7_ Axis 1 Loading
1	Col du Zad	2200m	Glueck and Stockton (2001)	984–1984	**0.698**	0.246	0.368
2	Atlas Mountains CO	2200m	Esper et al. (2007)	977–2001	**0.679**	0.306	0.432
3	Atlas Mountains TZ	2180m	Esper et al. (2007)	987–2001	**0.667**	0.309	0.411
4	Atlas Mountains CS	2200m	Esper et al. (2018)	1776–2001	**0.628**	0.287	–
5	Tounfite	2200m	Glueck and Stockton (2001)	1253–1984	**0.491**	0.230	0.354
6	Tadlounte Recollection	1920m	Touchan et al. (2011)	1696–2004	**0.478**	0.381	–
7	Ta'Adlount	2200m	Stockton et al. (2018a)	1728–1984	**0.474**	0.343	–
8	Atlas Mountains TO	2100m	Esper et al. (2007)	1271–2001	**0.429**	0.349	0.295
9	Atlas Mountains IS	1830m	Esper et al. (2007)	1784–2001	**0.424**	0.185	–
10	Ifrane	1900m	Chbouki et al. (2018a)	1549–1984	**0.383**	0.176	–
11	Atlas Mountains JF	2200m	Esper et al. (2007)	1021–2001	**0.308**	0.270	0.378
12	Tizi u Treten	1900m	Touchan et al. (2011)	1490–2003	**0.283**	0.139	–
13	Afechtal	1700m	Stockton et al. (2018b)	1632–1984	0.052	0.085	–
14	Tazzeka	1900m	Chbouki et al. (2018b)	1845–1984	0.029	−0.029	–
15	Tissouka	1700m	Chbouki et al. (2018c)	1748–1984	−0.007	0.054	–
16	Lake Sidi Ali	2200m	*This study*	1150-2013	na	0.250	0.394

M.a.s.l = meters above sea level. ^*^Values in bold typeface indicate correlation significant at p < 0.05.

The PCA analyses confirm a common dominant growth signal from the Middle and High Atlas. The first principal components of the long (PCA_7_; 1271–1984 CE) and short (PCA_16_; 1845–1984 CE) chronologies explain 55.7% and 40.1% of the variance, respectively (Supplementary Table 1). The sample scores on the first principal components are plotted against time in Fig. 2b and are nearly identical for PCA_7_ and PCA_16_ over the common time interval. For PCA_7_, only the first component is significant against the broken stick model of random variance. For PCA_16_, the second and third components are also significant, explaining an additional 17.8% and 12.8% of the variance, respectively. For PCA_7_, all sites (located in the southern Middle and High Atlas) have positive loadings and are positively correlated with the first component (Table 1, Fig. 3c). For PCA_16_, sites in the Middle and High Atlas have strong positive loadings and display positive correlations with the first component (Table 1, Fig. 3b), while the Rif Atlas sites have weak positive loadings and correlations, and one site located in the northeastern Middle Atlas displays a weak negative loading and negative correlation. Overall, these analyses confirm the dominant coherent climate-driven response of *C. atlantica* tree-rings in the old growth forests of the Middle and High Atlas. There are strong similarities between the Sidi Ali TRI and first principal component scores in terms of multidecadal shifts between above- and below-average growth (11 yr means and yellow shading, Fig. 2b). Given the near identical first principal component signals from the two regional growth signals (PCA_7_ and PCA_16_), we utilize the signal in the long chronologies (PCA_7_) for all further timeseries analyses. The second and third components of the short chronologies were not considered further, since they likely reflect subordinate but meaningful aspects of intra-regional growth responses.

### 3.3 Timeseries analyses

**Fig. 4:**
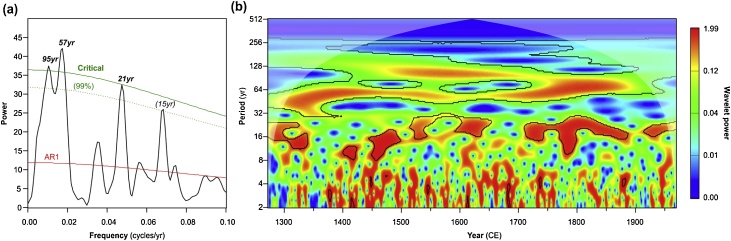
Results of Redfit and wavelet analyses of the *C. atlantica* PCA_7_ showing (a) Redfit power spectrum for decadal to centennial frequencies (frequency < 0.1 cycles per year) annotated with the AR1 model (red line), 99% confidence level (dotted green line) and critical false alarm level (99.8%, solid green line); spectral peaks exceeding the critical false alarm level are indicated in bold typeface with the corresponding period (yr); (b) wavelet power spectrum, with 95% significance limit (black lines) and the cone of influence (white shading). (For interpretation of the references to colour in this figure legend, the reader is referred to the web version of this article).

RedFit spectral analysis confirms the presence of significant periodic components inconsistent with a red noise (AR1) origin in the PCA_7_ (Fig. 4a). The dominant spectral peaks (exceeding the 99.8% critical false alarm level) are in the multidecadal frequency bands (<0.1 cycles per year) centered at 95 yr, 57 yr and 21 yr. The highest single spectral power is evidenced for the 57 yr period. At higher frequencies (>0.1 cycles per year), significant peaks are detected in the interannual (2–3 yr) frequencies. The wavelet analysis reveals the expression of the periodic components over time (Fig. 4b). The lower frequency multidecadal component (˜95 yr) is significant between 1400 and 1750 CE and reaches maximum power between 1500 and 1600 CE. It is not stationary but appears to be situated within a band of spectral power in the centennial to multidecadal range that gradually increases in frequency through the studied time interval. The multidecadal component (57 yr) is clearly expressed and significant throughout the whole time series. The higher frequency multidecadal (strictly, bidecadal) component corresponding to the RedFit spectral peak of 21 yr is episodically significant throughout the record and is most evident from 1700 to 1900 CE. Overall, the wavelet analysis of PCA_7_ highlights the strong and fairly stable expression of multidecadal oscillations between 1271 and 1984 CE.

**Fig. 5:**
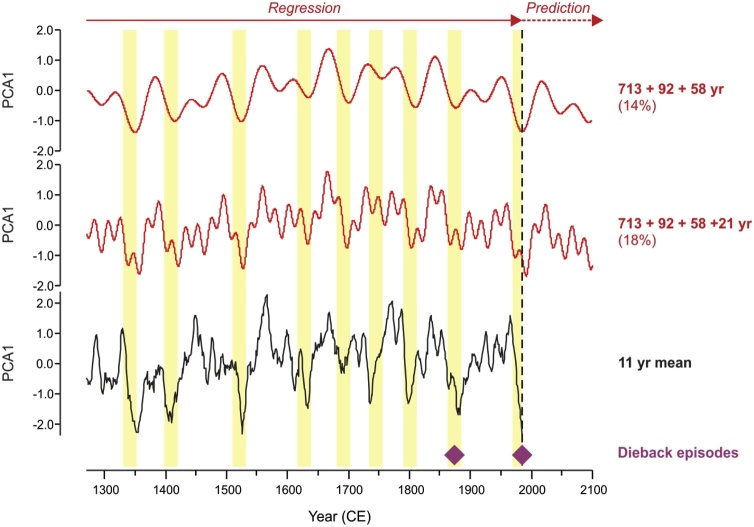
Sinusoidal regression modelling of the raw (annual, non-smoothed) *C. atlantica* PCA_7_ scores showing the composite models for (a) three and (b) four components. Models are labelled with the sinusoid periods (yr) and percentage of variance explained (100 x R^2^). All models are highly significant (p < 0.01). Modelled PCA_7_ components are projected forward to 2100 CE. The 11 yr mean PCA_7_ is shown for comparison (c), with yellow bars highlighting good correspondence between low growth (negative PCA_7_ scores, cf. Fig. 2) and regular declines in the composite models. Documented dieback phases (d) are indicated with purple diamonds. (For interpretation of the references to colour in this figure legend, the reader is referred to the web version of this article).

Modelling of significant periodicities for PCA_7_ indicates four best-fit significant sine components, explaining 4–5% of the variance each: 713 yr (5%), 92 yr (4%), 58 yr (5%) and 21 yr (4%). Composite models of three (713, 92 and 58 yr) and four (713, 92, 58, and 21 yr) terms (Fig. 5) capture 14% and 18% of the variance explained, respectively. The explanatory power for low frequency variability in PCA_7_ is much higher; the four-term composite explains 42% of the variance in the 11 yr smoothed PCA_7_ while the three-term composite explains 49% of the variance in the 21 yr PCA_7_. Overall, sinusoidal modelling provides an independent confirmation of the frequency, stability and significance of multidecadal periodic components in PCA_7._ Periods of decline in these composite models show good correspondence with low growth, including the documented dieback episodes in the 1870s–1880s and 1970s (Fig. 5). Forward modelling of the significant composite models suggests recovery from the current dieback episode over the next two decades, with a peak centered in recovery at ˜2030 CE.

### 3.4 Regional climatology

**Fig. 6:**
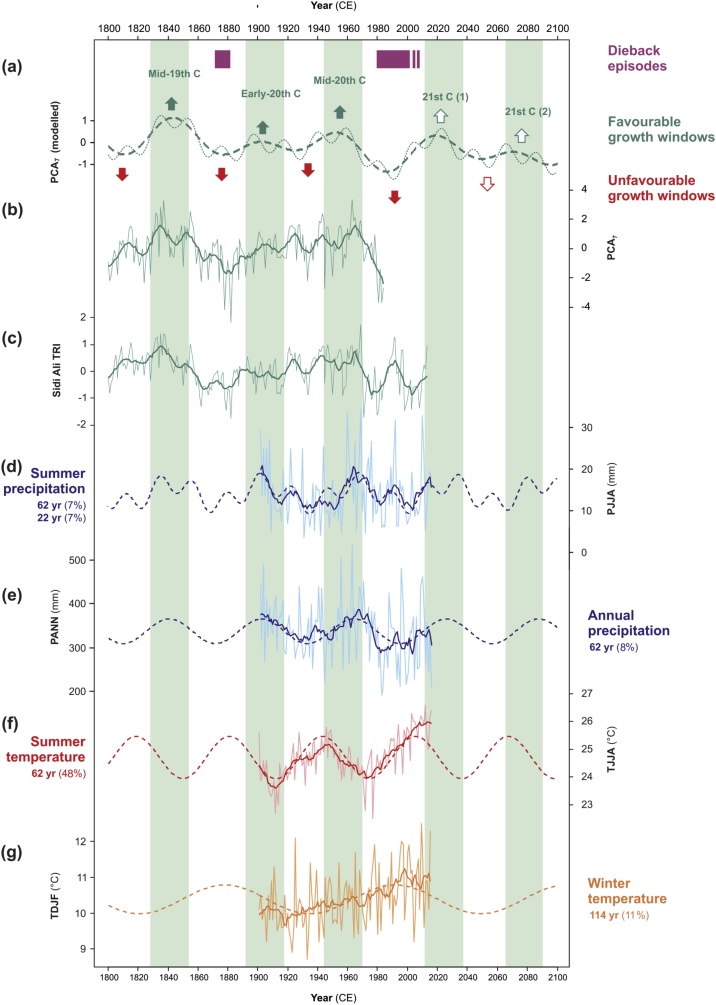
Exploration of oscillatory behavior in *C. atlantica* growth and regional climatology with inferences for growth conditions in the 21 st century, showing: (a) 3-component (dashed line) and 4-component (stippled line) sinusoidal models for PCA_7_ (cf. Fig. 5); (b) annual and 11 yr mean (bold line) PCA_7_; (c) annual and 11 yr mean (bold line) Sidi Ali TRI; (d) summer precipitation for Morocco with 11 yr mean (bold line) and 2-component best-fit sinusoidal model (dashed line); (e) annual precipitation for Morocco with 11 yr mean (bold line) and best-fit sinusoidal model (dashed line); (f) mean summer temperature for Morocco with 11 yr means (bold line) and best-fit sinusoidal model (dashed line); (g) mean winter temperature for Morocco, with 11 yr means (bold line) and best-fit sinusoidal model (dashed line). Climate data (d–g) from Harris et al. (2014). Green vertical shading highlights past and projected favourable growth windows corresponding to the ˜60 yr multidecadal oscillation in tree growth and climate. Intervening unfavourable growth windows (red arrows) include the documented historical dieback episodes (purple bars). (For interpretation of the references to colour in this figure legend, the reader is referred to the web version of this article).

For the historical climate interval 1901–1984 CE, PCA_7_ is positively correlated with annual precipitation (r = 0.55, p < 0.05) and weakly but significantly correlated to seasonal precipitation from the preceding autumn and winter through current spring and summer (Table 2). Annual and seasonal temperatures are not significantly correlated with PCA_7_. Sinusoidal regression modelling reveals significant fits for the historical precipitation data at periods of 62–64 yr (annual, spring and summer precipitation) and 22 yr (summer precipitation), explaining 7–8% of the variance in each case (Table 2). The amplitude of the ˜60 yr best-fit sinusoid for annual precipitation is ±28 mm, and the amplitude of the combined 62 yr and 22 yr sinusoids for summer precipitation is ±5 mm (Fig. 6d, e). For the temperature data, significant sinusoids at periods of 60–62 yr are evident in mean annual temperature as well as mean spring, summer and autumn temperatures, explaining 48%, 39%, 48% and 23% of the variance, respectively (Table 2). The amplitude of the ˜60 yr sinusoid is ±0.6 °C for annual temperatures and ±0.8 °C for summer temperatures (Fig. 6f). A sinusoid with a period of 114 yr is also significant for the annual, winter and autumn temperatures, explaining 11%, 13% and 5% of the variance respectively, with an amplitude of ±0.4 °C for winter temperatures (Fig. 6f).

**Table 2:** Results of correlation analysis and sinusoidal regression of regional climatology data for Morocco (Harris et al., 2014).

Variable	Season	Correlation against PCA_7_*	Significant sinusoids (*R*^2^)*		
			Centennial	Multidecadal	Bidecadal
Precipitation	Annual^a^	**0.55**	–	**62 yr** (*0.08*)	–
	Summer, preceding (JJA)	0.06	na	na	na
	Autumn, preceding (SON)	**0.26**	na	na	na
	Winter (DJF)	**0.32**	–	–	–
	Spring (MAM)	**0.23**	–	**64 yr** (*0.07*)	–
	Summer (JJA)	**0.29**	–	**62 yr** (*0.07*)	**22 yr** (*0.07*)
	Autumn (SON)	0.06	–	–	–
Temperature	Annual^a^	0.05	**114 yr** (*0.11*)	**62 yr** (*0.48*)	–
	Summer, preceding (JJA)	0.15	na	na	na
	Autumn, preceding (SON)	0.02	na	na	na
	Winter (DJF)	0.06	**114 yr** (*0.13*)	–	–
	Spring (MAM)	−0.03	–	**60 yr** (*0.39*)	–
	Summer (JJA)	0.06	–	**62 yr** (*0.48*)	–
	Autumn (SON)	−0.13	**114 yr** (*0.05*)	**62 yr** (*0.23*)	–

*Values in bold typeface indicate correlation significant at p < 0.05.

a Preceding September to August.

The ˜60 yr periodic components of the PCA_7_ and annual precipitation are closely aligned for the timespan of the climate data, with minima in the modelled PCA_7_ corresponding to 60 yr minima in precipitation centered on the 1930s and 1990s (Fig. 6a). Extending the sine waves backwards to 1800 CE and forwards to 2100 CE highlights the close in-phase relationship between periodic components of PCA_7_, annual and summer precipitation, although the measurement period for PANN is short (approximately two cycles). The bidecadal (˜22 yr) fluctuations in PCA_7_ and the Lake Sidi Ali TRI with summer precipitation are also closely aligned, both for the recent decades documented in the new Lake Sidi Ali chronology (Fig. 6c) and for the backward projection of the sinusoidal model through the mid-19^th^ Century (Fig. 6d). Fig. 6 also shows that minima in the PCA_7_ precipitation models correspond to the rising limb of the ˜60 yr regional temperature cycle, which is out-of-phase with PANN by around a quarter cycle. We note that documented dieback episodes in the late 19^th^ and late 20^th^ centuries (Fig. 6a) correspond with unfavourable growth windows characterized by extended intervals of below average tree ring widths, minima in the modelled PANN cycle (Fig. 6e), the rising limb of the modelled annual temperature cycle (Fig. 6f), as well as higher winter temperatures (Fig. 6g). Finally, forward projection of the PCA_7_ and climate variables suggests two favourable growth windows for the 21st century (Fig. 6a), extending from present until 2040 CE, and from 2070 to 2090 CE.

## 4 Discussion

Previous studies highlight the value of *C. atlantica* tree-ring records for understanding past climatic variability on a range of timescales. This study presents several novel features of the *C. atlantica* archive, enhanced by a new chronology from the sensitive old-growth forests of the Middle Atlas, Morocco. First, the regionally coherent *C. atlantica* growth signal in the Middle and High Atlas contains periodic multidecadal fluctuations (95 yr, 57 yr, 21 yr) reflected in recurrent low-growth intervals between 1271 and 1984 CE, providing a long-term context for the current dieback episode. Second, these multidecadal fluctuations have parallels in historical regional precipitation and temperature variability, which yield insights into the climatic drivers of tree growth. Finally, forward projection of modelled periodic components in both the *C. atlantica* growth and regional climatology data allows for the evaluation of future growth trends for the next century, highlighting the importance of current management actions to minimise non-climatic stressors on *C. atlantica* in the Middle and High Atlas.

The coherence between *C. atlantica* growth at Lake Sidi Ali with other *C. atlantica* from the Middle and High Atlas portion of its range suggests a common regional driver of tree growth. This is confirmed by the robust and pervasive multidecadal growth fluctuations (95 yr, 57 yr, 21 yr) exhibited in the common long-term signal (PCA_7_). Previous studies have identified drought impacts on *C. atlantica* growth across a range of timescales, and in many cases these are consistent with the multidecadal periodic components identified here. Chbouki et al. (1995) report a 20–25 yr alternation of wet-dry intervals since the mid-19th century, with drought conditions in 1860–1890 CE, 1925–1950 CE and in the 1970s. These dry intervals correspond to minima in the ˜60 yr oscillation in tree growth and precipitation identified in this study (Fig. 6), and hence are consistent with growth fluctuations occurring over much longer timescales since at least 1300 CE. With a focus on annual extremes, Kherchouche et al. (2012) infer an unusually intense drought in the years 1877–79 CE and 1977–78 CE in Algeria, again consistent with periodic minima in the common growth signal presented here. This good match suggests both a possible association of short-term drought extremes and long-term growth minima oscillations. This coherency also points to geographic parallels in *C. atlantica* growth patterns beyond Morocco, which are not currently testable based on publicly available data in the ITRDB.

This study places the growth decline and widespread dieback of *C. atlantica* since the 1970s in a longer-term context. This decline is not a unique feature of the recent tree-ring record (see also Esper et al., 2007; Touchan et al., 2011), and is in fact consistent with long-term patterns of drought in the western Mediterranean region (Cook et al., 2016). Although recent anthropogenic and biotic pressures may be enhancing the decline and resulting in reduced *C. atlantica* regeneration (Benabid and Fennane, 1994; Quézel, 2000; Abdelhamid et al., 2017), low growth intervals of comparable duration have occurred multiple times in the past 850 years. These intervals appear consistent with long-term oscillations on multidecadal (95 yr, ˜60 yr, and 21 yr) timescales (Fig. 5), and hence broadly predictable with respect to historical patterns (and without recourse to unique global change drivers of the 20th century). The Lake Sidi Ali chronology is based on trees that have survived (rather than succumbed) to this variability, and it is likely that episodes of increased tree mortality occurred throughout the period covered by the *C. atlantica* chronologies (cf. Cailleret et al., 2017). Indeed, the historical dieback event of 1872–1882 CE (Bentouati, 2008; Kherchouche et al., 2012; Touchan et al., 2008) corresponds to an interval of low growth associated with the multidecadal oscillations (Fig. 5, Fig. 6), specifically the previous and penultimate minima in the 95 yr and 56 yr fluctuations, respectively. Consistent with regular, slow-changing fluctuations, the updated chronology provided by the Lake Sidi Ali *C. atlantica* (extending to 2013 CE) indicates that growth may be recovering from drought commencing in the 1970s, consistent with the pervasive 56 yr fluctuation, while a second interval of recent low growth experienced in the early 2000s is consistent with bidecadal (˜21 yr) fluctuations.

A major challenge in understanding vegetation response to climate in semi-arid regions is disentangling the role of temperature and precipitation changes in creating drought stress. Our analysis of the common signal (PCA_7_) in long Middle and High Atlas *C. atlantica* chronologies and regional climatology points to a significant relationship with annual precipitation as well as positive relationships with seasonal precipitation from the preceding autumn to current summer (Table 2). Previous studies have identified preceding autumn or winter precipitation as having the strongest relationship with *C. atlantica* growth (*e.g.*, Till, 1987; Till and Guiot, 1990), which underpins the subsequent reconstruction of the North Atlantic Oscillation using *C. atlantica* records (Trouet et al., 2009). Other work emphasizes the role of spring and summer drought stress (Esper et al., 2007; Linares et al., 2013) as reflected in the scanning Palmer Drought Stress Severity (scPDSI) index. Tree-ring sensitivity to different climatological drivers may vary from site to site and between age cohorts (Linares et al., 2013), and exploration of inter- and intra-site tree growth responses is beyond the scope of the current study. However, our analysis points to annual precipitation as the dominant driver of common year-to-year variability across the Middle and High Atlas ranges. At multidecadal scales, only spring and summer season precipitation show significant variability at the dominant ˜60 yr period observed in the tree-ring data, and only summer precipitation shows significant fluctuations at both ˜60 yr and ˜21 yr (Table 2, Fig. 6). This suggests that low frequency fluctuations in summer precipitation of modest amplitude (±10 mm) may have a strong influence on growing season conditions and tree growth at decadal and greater timescales. The apparent high sensitivity to slow-changing background levels of spring and summer precipitation may reside in the previously reported drought-tolerating physiological mechanisms of *C. atlantica*, which allow it to maintain growth under severely water-stressed conditions but leave it vulnerable to extended (inter-annual) drought (Aussenac, 1984). Our findings are consistent with the implication of low spring precipitation in recent dieback of *C. atlantica* in Morocco (Rhanem, 2011).

The absence of significant correlations between PCA_7_ and annual or seasonal temperatures suggests that growth response to temperatures may be conditioned by site factors or may be less important for short-term growth. Nevertheless, the strength and high signal-to-noise ratio of multidecadal fluctuations in regional temperatures (Table 2, Fig. 6) suggest that interactions between precipitation and temperature are important in controlling long-term growth conditions. In particular, the ˜60 yr oscillation in spring, summer and autumn temperatures may exacerbate moisture stress during the growing season by increasing evaporative demand at times of low precipitation. In addition, a slow-changing component of winter temperature variability may help to explain the timing of the most recent dieback episode. Winter temperatures are highlighted as an important control on long-term health and regeneration of *C. atlantica* forests (Cheddadi et al., 2017). In this case, the near-centennial (˜115 yr) periodicity of change in winter temperature can only be tentatively identified due to the timespan of the historical data, but it may help to explain both the severity of the recent and late 19th century dieback episodes in terms of a compound influence of sub-optimal (warm) winter conditions and low growing season moisture availability.

Over the timespan of the regional climatological data (1901–2016), the strongest multidecadal fluctuation of ˜60 yr in tree growth has counterparts in both precipitation and temperature variability. Although the timespan of the climatological data is relatively short, extrapolation of the ˜60 yr component highlights that regularities in the slow-changing background climate may underpin the pervasive multidecadal fluctuations in *C. atlantica* growth, i.e. a combination of reduced precipitation and increasing temperatures driving bioclimatic drought stress. The ˜60 yr oscillation is evident in growing season climate (spring and summer precipitation, spring to autumn temperatures) but is not evident in winter season precipitation or temperatures. Multidecadal variability (˜60 yr cycle) in tropical and sub-tropical precipitation has been reported in long records of the Indian monsoon (Agnihotri et al., 2002) and Arabian Sea precipitation (von Rad et al., 1999), and is also a significant feature of Pacific climate variability (*e.g.* D’Aleo and Easterbrook, 2010). Multidecadal temperature variability is also well known in the Atlantic Multidecadal Oscillation (Enfield et al., 2001). These parallels suggest that the ˜60 yr oscillation may originate in low-latitude climate variability with a prevailing signature over northwest Africa from spring to autumn. The 21 yr oscillation in tree growth for which we only find a regional climatological counterpart in summer precipitation further supports a summer-biased climatic signal. Although a ˜60 yr fluctuation in the North Atlantic Oscillation has been previously reported (Mazzarella and Scafetta, 2012), we do not find support for this pattern in the winter precipitation data. However, a slow-changing component of winter temperatures is evident in the Moroccan dataset which may modulate tree growth and contribute to the near-centennial spacing of recent dieback episodes (Fig. 6f). Further research is required to understand whether the climatic drivers of tree growth fluctuations reside in low- or high-latitude (summer *vs.* winter) climate variability and elucidate the teleconnections involved.

Observations of historical growth patterns and the model-based projections (Fig. 6) suggest that climatic windows for improved growth could be anticipated in the 21st century. In particular, the low-frequency components of tree growth suggest that above average growth may occur until the 2040s and again near the end of the 21st century, corresponding to the next two maxima in the ˜60 yr cycle (21st century favourable growth windows (1) & (2), Fig. 6). Overall, based on projected growth trends, higher growth rates are anticipated throughout the 21st century, with a next likely window for major dieback in the early 22nd century, corresponding to modulation by the ˜95 yr cycle. If slow-changing periodic dynamics in the regional climate remain consistent through the coming century, they should mitigate, at least in the short-term, the poor prognosis for the survival of *C. atlantica* under climate change scenarios (Cheddadi et al., 2017). As such, the next decades will provide a critical opportunity for actions to conserve this endangered species, particularly in the Middle and High Atlas portions of its range represented in this study. However, this would also require mitigation of suppressed regeneration and anthropogenic impacts, including soil erosion, grazing, timber logging and firewood collection, which would otherwise counteract the recovery due to climate changes (McGregor et al., 2009; Trouet et al., 2009; Abel-Schaad et al., 2018). Although resilience of the *C. atlantica* to historical droughts is impressive, our analyses highlight the urgency of regional conservation actions to ensure local populations are maintained and renewed through the current period of climate-induced stress (Cheddadi et al., 2017; Départment des forêts, 2010; Linares et al., 2013) and capitalise on the potential upcoming windows of climatic opportunity.

## 5 Conclusions

Conservation of the endangered *C. atlantica* is a priority throughout its North African range, where it has exhibited widespread dieback since the 1970s. In this study, we provide long-term context for this dieback and explore climatological variability driving growth fluctuations through time. A regionally-coherent growth signal from the Middle and High Atlas reveals multidecadal periodicity reflecting recurrent low-growth intervals from 1271 to 1984 CE. These multidecadal fluctuations originate primarily in growing season (spring and summer) precipitation variability, compounded by slow-changing components of summer and winter temperature. Modelled growth patterns and climate variables suggest that the 1970s dieback is consistent with historical climatically-driven growth declines, and forward projection of this model suggests two periods of favourable growth are likely in the 21st century. We anticipate that these favourable growth periods may enhance current conservation actions targeting the Middle and High Atlas populations of the *C. atlantica* and suggest efforts are made to minimise non-climatic stressors on this endangered species to support its long-term survival.

## Conflict of interest

The authors declare no conflicts of interest in the preparation or publication of this manuscript.

## Data accessibility statement

Upon acceptance of publication, the Sidi Ali chronology will be uploaded to NOAA’s International Tree-Ring Databank (https://www.ncdc.noaa.gov/data-access/paleoclimatology-data/datasets/tree-ring), the world’s largest repository for tree-ring data sets.
