# Supplementary material for: Multidecadal variability in Atlas cedar growth in Northwest Africa during the last 850 years: Implications for dieback and conservation of an endangered species
Source: Dendrochronologia (Verona). 2019 Aug;56:125599. doi: 10.1016/j.dendro.2019.05.003 (PMC6686623; doi:10.1016/j.dendro.2019.05.003)
Supplement: Supplementary file 1 [file mmc1.docx]

**Multidecadal variability in Atlas cedar growth in Northwest Africa during the last 850 years: Implications for dieback and conservation of an endangered species**

Kelsey Copes-Gerbitz, William Fletcher, Jonathan G.A. Lageard, Mustapha Rhanem, Sandy P. Harrison

**Supplementary Table 1**

Timeseries datasets shown in main text Figure 2.

| **Year CE** | **Sidi Ali TRI** | **PCA_7_** | **PCA_16_** |
| --- | --- | --- | --- |
| 1150 | 1.217 | NA | NA |
| 1151 | 0.249 | NA | NA |
| 1152 | 1.575 | NA | NA |
| 1153 | 1.197 | NA | NA |
| 1154 | 0.575 | NA | NA |
| 1155 | 1.254 | NA | NA |
| 1156 | 0.840 | NA | NA |
| 1157 | 2.227 | NA | NA |
| 1158 | -0.509 | NA | NA |
| 1159 | 1.645 | NA | NA |
| 1160 | -0.004 | NA | NA |
| 1161 | -1.779 | NA | NA |
| 1162 | -0.760 | NA | NA |
| 1163 | 0.047 | NA | NA |
| 1164 | -0.517 | NA | NA |
| 1165 | 0.163 | NA | NA |
| 1166 | 0.354 | NA | NA |
| 1167 | 0.200 | NA | NA |
| 1168 | 0.971 | NA | NA |
| 1169 | 0.389 | NA | NA |
| 1170 | 0.743 | NA | NA |
| 1171 | -1.435 | NA | NA |
| 1172 | -1.402 | NA | NA |
| 1173 | 1.165 | NA | NA |
| 1174 | 1.111 | NA | NA |
| 1175 | 0.643 | NA | NA |
| 1176 | 0.661 | NA | NA |
| 1177 | 1.139 | NA | NA |
| 1178 | 0.989 | NA | NA |
| 1179 | 0.355 | NA | NA |
| 1180 | 1.240 | NA | NA |
| 1181 | 1.569 | NA | NA |
| 1182 | -0.551 | NA | NA |
| 1183 | 1.109 | NA | NA |
| 1184 | 1.260 | NA | NA |
| 1185 | 0.943 | NA | NA |
| 1186 | -0.664 | NA | NA |
| 1187 | 0.014 | NA | NA |
| 1188 | -0.147 | NA | NA |
| 1189 | -1.296 | NA | NA |
| 1190 | -0.116 | NA | NA |
| 1191 | 0.154 | NA | NA |
| 1192 | -0.078 | NA | NA |
| 1193 | 0.168 | NA | NA |
| 1194 | 0.140 | NA | NA |
| 1195 | 0.658 | NA | NA |
| 1196 | -1.838 | NA | NA |
| 1197 | -1.915 | NA | NA |
| 1198 | -2.386 | NA | NA |
| 1199 | 0.059 | NA | NA |
| 1200 | 0.184 | NA | NA |
| 1201 | 0.085 | NA | NA |
| 1202 | -0.338 | NA | NA |
| 1203 | -0.482 | NA | NA |
| 1204 | 0.255 | NA | NA |
| 1205 | 1.312 | NA | NA |
| 1206 | 0.360 | NA | NA |
| 1207 | 1.356 | NA | NA |
| 1208 | 1.438 | NA | NA |
| 1209 | 0.948 | NA | NA |
| 1210 | 1.205 | NA | NA |
| 1211 | 1.191 | NA | NA |
| 1212 | 0.844 | NA | NA |
| 1213 | -0.003 | NA | NA |
| 1214 | 1.294 | NA | NA |
| 1215 | -0.401 | NA | NA |
| 1216 | -0.193 | NA | NA |
| 1217 | -0.037 | NA | NA |
| 1218 | -0.291 | NA | NA |
| 1219 | 0.456 | NA | NA |
| 1220 | -1.419 | NA | NA |
| 1221 | -0.177 | NA | NA |
| 1222 | -0.539 | NA | NA |
| 1223 | 1.357 | NA | NA |
| 1224 | 1.082 | NA | NA |
| 1225 | 1.669 | NA | NA |
| 1226 | 0.254 | NA | NA |
| 1227 | 0.866 | NA | NA |
| 1228 | -0.173 | NA | NA |
| 1229 | 0.732 | NA | NA |
| 1230 | 0.977 | NA | NA |
| 1231 | 0.950 | NA | NA |
| 1232 | 1.095 | NA | NA |
| 1233 | 1.026 | NA | NA |
| 1234 | -1.333 | NA | NA |
| 1235 | 1.523 | NA | NA |
| 1236 | 1.539 | NA | NA |
| 1237 | -0.080 | NA | NA |
| 1238 | 1.755 | NA | NA |
| 1239 | 0.979 | NA | NA |
| 1240 | 0.986 | NA | NA |
| 1241 | 1.403 | NA | NA |
| 1242 | -1.540 | NA | NA |
| 1243 | -1.702 | NA | NA |
| 1244 | 0.008 | NA | NA |
| 1245 | 1.087 | NA | NA |
| 1246 | 0.630 | NA | NA |
| 1247 | -0.360 | NA | NA |
| 1248 | -0.313 | NA | NA |
| 1249 | -1.679 | NA | NA |
| 1250 | -0.686 | NA | NA |
| 1251 | -0.113 | NA | NA |
| 1252 | 0.075 | NA | NA |
| 1253 | -0.533 | NA | NA |
| 1254 | -0.172 | NA | NA |
| 1255 | -0.344 | NA | NA |
| 1256 | -1.012 | NA | NA |
| 1257 | -1.025 | NA | NA |
| 1258 | -1.580 | NA | NA |
| 1259 | -1.065 | NA | NA |
| 1260 | -1.003 | NA | NA |
| 1261 | -0.658 | NA | NA |
| 1262 | -1.282 | NA | NA |
| 1263 | -1.051 | NA | NA |
| 1264 | -1.403 | NA | NA |
| 1265 | -1.166 | NA | NA |
| 1266 | -2.016 | NA | NA |
| 1267 | -0.659 | NA | NA |
| 1268 | -0.554 | NA | NA |
| 1269 | -0.453 | NA | NA |
| 1270 | -0.226 | NA | NA |
| 1271 | -0.999 | -0.146 | NA |
| 1272 | -1.108 | 0.106 | NA |
| 1273 | -1.267 | -0.293 | NA |
| 1274 | -1.620 | -0.774 | NA |
| 1275 | -0.557 | -0.966 | NA |
| 1276 | -0.721 | -1.004 | NA |
| 1277 | -1.034 | -0.601 | NA |
| 1278 | -1.523 | -1.144 | NA |
| 1279 | -0.947 | -1.337 | NA |
| 1280 | -0.767 | -0.597 | NA |
| 1281 | -0.748 | -0.854 | NA |
| 1282 | 0.482 | 0.654 | NA |
| 1283 | 0.197 | 0.093 | NA |
| 1284 | 1.310 | 1.302 | NA |
| 1285 | 0.335 | 1.181 | NA |
| 1286 | 1.382 | 2.593 | NA |
| 1287 | 0.740 | 2.108 | NA |
| 1288 | 0.364 | 1.146 | NA |
| 1289 | 0.589 | 1.151 | NA |
| 1290 | 0.599 | 0.439 | NA |
| 1291 | 0.824 | 0.161 | NA |
| 1292 | 0.316 | -0.583 | NA |
| 1293 | -0.819 | -1.493 | NA |
| 1294 | -1.670 | -2.161 | NA |
| 1295 | -0.492 | -1.084 | NA |
| 1296 | 0.313 | -0.412 | NA |
| 1297 | -0.267 | 0.079 | NA |
| 1298 | -0.913 | -0.790 | NA |
| 1299 | -1.541 | -1.545 | NA |
| 1300 | -1.205 | -1.751 | NA |
| 1301 | -0.028 | -1.021 | NA |
| 1302 | 0.562 | -0.517 | NA |
| 1303 | -0.021 | -0.050 | NA |
| 1304 | 0.897 | 0.297 | NA |
| 1305 | 1.334 | 1.173 | NA |
| 1306 | 1.488 | 0.850 | NA |
| 1307 | 0.882 | -0.317 | NA |
| 1308 | 0.264 | -0.945 | NA |
| 1309 | -1.608 | -3.119 | NA |
| 1310 | -0.433 | -0.821 | NA |
| 1311 | 0.010 | -1.183 | NA |
| 1312 | 0.027 | -1.196 | NA |
| 1313 | 0.177 | -1.122 | NA |
| 1314 | 1.011 | 0.628 | NA |
| 1315 | 0.163 | -0.760 | NA |
| 1316 | 0.206 | -0.449 | NA |
| 1317 | 0.759 | -0.049 | NA |
| 1318 | 1.457 | 0.170 | NA |
| 1319 | 1.203 | 0.097 | NA |
| 1320 | 0.094 | -0.872 | NA |
| 1321 | -0.022 | -1.878 | NA |
| 1322 | 0.002 | -0.917 | NA |
| 1323 | 0.710 | -0.709 | NA |
| 1324 | -1.854 | -3.205 | NA |
| 1325 | 1.526 | 1.514 | NA |
| 1326 | 0.974 | 0.374 | NA |
| 1327 | 0.957 | 0.785 | NA |
| 1328 | 1.340 | 1.283 | NA |
| 1329 | 2.052 | 2.455 | NA |
| 1330 | 2.304 | 2.717 | NA |
| 1331 | 2.353 | 2.631 | NA |
| 1332 | 1.732 | 2.836 | NA |
| 1333 | 1.182 | 1.559 | NA |
| 1334 | -1.192 | -1.915 | NA |
| 1335 | -0.331 | -1.239 | NA |
| 1336 | 1.106 | -0.225 | NA |
| 1337 | -1.445 | -2.107 | NA |
| 1338 | 1.517 | -0.161 | NA |
| 1339 | 0.858 | -0.453 | NA |
| 1340 | 0.479 | -0.598 | NA |
| 1341 | -0.611 | -2.150 | NA |
| 1342 | -0.503 | -1.779 | NA |
| 1343 | -0.121 | -1.205 | NA |
| 1344 | -0.633 | -1.023 | NA |
| 1345 | -0.693 | -1.025 | NA |
| 1346 | -1.055 | -1.215 | NA |
| 1347 | -1.323 | -2.075 | NA |
| 1348 | -1.560 | -2.753 | NA |
| 1349 | -0.736 | -2.283 | NA |
| 1350 | -0.911 | -2.288 | NA |
| 1351 | -0.946 | -2.717 | NA |
| 1352 | -0.852 | -2.722 | NA |
| 1353 | -0.579 | -1.835 | NA |
| 1354 | -0.554 | -1.666 | NA |
| 1355 | -0.677 | -2.155 | NA |
| 1356 | -0.959 | -2.520 | NA |
| 1357 | -0.763 | -2.253 | NA |
| 1358 | -0.706 | -2.389 | NA |
| 1359 | -1.416 | -2.850 | NA |
| 1360 | -0.842 | -1.962 | NA |
| 1361 | -0.359 | -1.408 | NA |
| 1362 | 0.042 | -0.437 | NA |
| 1363 | -0.608 | -1.139 | NA |
| 1364 | -0.014 | -1.429 | NA |
| 1365 | 0.304 | 0.067 | NA |
| 1366 | 0.114 | -0.604 | NA |
| 1367 | 0.041 | -0.771 | NA |
| 1368 | 0.814 | 0.555 | NA |
| 1369 | 0.851 | 0.194 | NA |
| 1370 | 0.391 | -0.796 | NA |
| 1371 | 0.109 | -0.284 | NA |
| 1372 | 0.529 | -0.656 | NA |
| 1373 | 0.193 | -1.321 | NA |
| 1374 | 0.344 | 0.303 | NA |
| 1375 | 0.877 | 0.272 | NA |
| 1376 | -1.299 | -4.661 | NA |
| 1377 | 0.816 | 0.186 | NA |
| 1378 | 0.280 | -0.447 | NA |
| 1379 | -1.041 | -2.276 | NA |
| 1380 | 1.369 | 1.382 | NA |
| 1381 | 0.932 | 0.000 | NA |
| 1382 | 0.908 | -0.458 | NA |
| 1383 | 0.697 | -0.541 | NA |
| 1384 | -1.772 | -0.577 | NA |
| 1385 | -0.034 | -1.592 | NA |
| 1386 | 0.246 | -1.563 | NA |
| 1387 | 1.056 | 1.076 | NA |
| 1388 | 1.138 | 0.307 | NA |
| 1389 | -0.060 | -1.227 | NA |
| 1390 | 1.196 | 0.431 | NA |
| 1391 | 0.929 | -0.237 | NA |
| 1392 | 1.622 | 1.198 | NA |
| 1393 | 1.034 | 0.189 | NA |
| 1394 | 1.157 | 0.968 | NA |
| 1395 | -1.180 | -3.842 | NA |
| 1396 | 0.890 | -0.239 | NA |
| 1397 | 0.326 | 0.067 | NA |
| 1398 | 0.532 | 0.305 | NA |
| 1399 | 0.519 | 0.637 | NA |
| 1400 | -1.161 | -1.512 | NA |
| 1401 | -0.759 | -0.698 | NA |
| 1402 | -1.363 | -2.737 | NA |
| 1403 | -1.056 | -1.955 | NA |
| 1404 | -1.360 | -2.860 | NA |
| 1405 | -2.505 | -3.409 | NA |
| 1406 | -1.660 | -2.763 | NA |
| 1407 | -1.461 | -2.131 | NA |
| 1408 | -1.102 | -0.715 | NA |
| 1409 | -0.959 | -1.184 | NA |
| 1410 | -1.082 | -1.209 | NA |
| 1411 | -1.017 | -0.035 | NA |
| 1412 | -0.686 | 0.960 | NA |
| 1413 | -2.033 | -4.357 | NA |
| 1414 | -1.498 | -0.970 | NA |
| 1415 | -1.606 | -5.839 | NA |
| 1416 | -0.834 | -0.683 | NA |
| 1417 | -0.743 | -1.123 | NA |
| 1418 | -0.054 | -0.172 | NA |
| 1419 | 0.025 | 0.220 | NA |
| 1420 | 0.583 | 0.584 | NA |
| 1421 | 0.625 | 0.444 | NA |
| 1422 | -0.907 | -2.930 | NA |
| 1423 | 0.168 | 0.361 | NA |
| 1424 | -0.895 | -1.491 | NA |
| 1425 | -0.043 | -1.357 | NA |
| 1426 | -0.300 | -1.297 | NA |
| 1427 | 0.272 | 0.337 | NA |
| 1428 | -0.429 | -0.766 | NA |
| 1429 | -0.116 | 0.413 | NA |
| 1430 | -0.519 | -0.509 | NA |
| 1431 | 0.516 | 0.840 | NA |
| 1432 | 0.839 | 1.128 | NA |
| 1433 | 0.534 | 0.145 | NA |
| 1434 | -0.579 | -1.014 | NA |
| 1435 | 0.268 | 1.246 | NA |
| 1436 | 0.624 | 1.736 | NA |
| 1437 | 0.266 | 0.375 | NA |
| 1438 | -0.761 | -2.304 | NA |
| 1439 | -0.094 | -0.109 | NA |
| 1440 | -1.282 | -2.449 | NA |
| 1441 | 0.239 | 1.187 | NA |
| 1442 | -0.282 | 0.442 | NA |
| 1443 | 0.764 | 1.705 | NA |
| 1444 | 0.129 | 1.882 | NA |
| 1445 | -0.624 | -1.189 | NA |
| 1446 | 0.065 | 1.231 | NA |
| 1447 | 0.849 | 2.614 | NA |
| 1448 | 1.339 | 3.595 | NA |
| 1449 | -0.779 | -2.094 | NA |
| 1450 | 0.816 | 2.224 | NA |
| 1451 | 0.343 | 1.418 | NA |
| 1452 | 1.747 | 3.895 | NA |
| 1453 | 0.939 | 2.297 | NA |
| 1454 | 0.356 | 1.707 | NA |
| 1455 | 0.601 | 1.362 | NA |
| 1456 | -0.817 | -1.859 | NA |
| 1457 | -0.564 | -2.257 | NA |
| 1458 | 0.456 | 0.348 | NA |
| 1459 | -0.173 | -0.585 | NA |
| 1460 | 0.169 | 0.571 | NA |
| 1461 | 1.259 | 2.818 | NA |
| 1462 | 1.001 | 2.121 | NA |
| 1463 | 0.269 | 0.528 | NA |
| 1464 | 1.288 | 2.286 | NA |
| 1465 | -0.023 | -1.006 | NA |
| 1466 | 0.926 | -0.146 | NA |
| 1467 | 0.520 | -0.716 | NA |
| 1468 | -2.127 | -4.067 | NA |
| 1469 | -1.457 | -3.645 | NA |
| 1470 | -0.324 | -0.821 | NA |
| 1471 | 0.220 | 0.442 | NA |
| 1472 | 0.208 | -0.270 | NA |
| 1473 | -0.233 | 0.067 | NA |
| 1474 | 0.063 | -0.114 | NA |
| 1475 | 0.672 | 2.488 | NA |
| 1476 | 1.049 | 2.844 | NA |
| 1477 | 0.685 | 1.952 | NA |
| 1478 | 0.563 | 0.107 | NA |
| 1479 | 0.093 | 0.039 | NA |
| 1480 | 1.011 | 1.335 | NA |
| 1481 | 0.595 | 0.522 | NA |
| 1482 | 0.352 | 0.266 | NA |
| 1483 | 0.431 | -1.845 | NA |
| 1484 | -0.461 | -1.957 | NA |
| 1485 | -0.075 | -1.635 | NA |
| 1486 | 0.613 | -0.379 | NA |
| 1487 | 0.235 | -1.327 | NA |
| 1488 | 0.934 | 0.027 | NA |
| 1489 | 1.333 | 0.827 | NA |
| 1490 | 1.077 | 0.332 | NA |
| 1491 | 1.195 | 1.759 | NA |
| 1492 | 1.110 | 1.529 | NA |
| 1493 | -1.213 | -3.109 | NA |
| 1494 | 0.997 | 0.716 | NA |
| 1495 | 0.758 | 1.401 | NA |
| 1496 | 0.551 | 0.149 | NA |
| 1497 | 0.212 | -0.383 | NA |
| 1498 | -0.302 | -0.520 | NA |
| 1499 | 0.808 | 0.370 | NA |
| 1500 | -0.765 | -2.247 | NA |
| 1501 | 0.503 | -0.154 | NA |
| 1502 | -0.668 | -1.077 | NA |
| 1503 | 0.201 | 0.433 | NA |
| 1504 | 0.035 | 0.262 | NA |
| 1505 | -0.082 | 0.375 | NA |
| 1506 | -0.106 | 0.887 | NA |
| 1507 | 0.485 | 1.078 | NA |
| 1508 | -0.657 | -0.785 | NA |
| 1509 | -0.685 | -0.367 | NA |
| 1510 | -0.410 | -0.801 | NA |
| 1511 | 0.069 | -0.644 | NA |
| 1512 | -0.418 | -1.084 | NA |
| 1513 | 0.748 | 1.003 | NA |
| 1514 | 0.615 | 1.299 | NA |
| 1515 | -0.880 | -2.290 | NA |
| 1516 | 0.345 | 0.611 | NA |
| 1517 | -0.030 | 0.135 | NA |
| 1518 | -0.215 | 0.190 | NA |
| 1519 | 0.444 | 1.124 | NA |
| 1520 | 0.182 | 0.647 | NA |
| 1521 | -1.836 | -4.750 | NA |
| 1522 | -0.300 | -1.072 | NA |
| 1523 | 0.068 | -0.585 | NA |
| 1524 | 0.577 | -1.157 | NA |
| 1525 | -1.195 | -2.738 | NA |
| 1526 | -1.377 | -3.932 | NA |
| 1527 | -1.118 | -2.339 | NA |
| 1528 | -1.359 | -2.307 | NA |
| 1529 | -1.971 | -3.820 | NA |
| 1530 | -0.928 | -1.398 | NA |
| 1531 | -0.938 | -1.770 | NA |
| 1532 | -0.378 | 0.085 | NA |
| 1533 | -0.395 | -0.052 | NA |
| 1534 | -0.009 | 0.932 | NA |
| 1535 | -0.892 | -0.381 | NA |
| 1536 | -0.591 | 0.193 | NA |
| 1537 | -0.552 | 0.687 | NA |
| 1538 | -0.745 | -0.254 | NA |
| 1539 | -0.436 | 0.259 | NA |
| 1540 | -0.368 | 0.369 | NA |
| 1541 | -0.861 | -0.877 | NA |
| 1542 | -0.957 | -1.956 | NA |
| 1543 | -0.007 | 0.555 | NA |
| 1544 | 0.433 | 1.106 | NA |
| 1545 | -1.420 | -3.757 | NA |
| 1546 | -0.006 | 0.496 | NA |
| 1547 | 0.816 | 1.289 | NA |
| 1548 | 0.332 | 0.505 | NA |
| 1549 | -0.680 | -0.890 | NA |
| 1550 | -0.009 | 0.611 | NA |
| 1551 | -0.039 | 0.348 | NA |
| 1552 | 0.202 | 0.684 | NA |
| 1553 | 0.796 | 1.437 | NA |
| 1554 | 1.286 | 2.458 | NA |
| 1555 | 0.833 | -0.472 | NA |
| 1556 | 1.367 | 2.605 | NA |
| 1557 | 0.576 | 0.855 | NA |
| 1558 | 0.716 | 0.633 | NA |
| 1559 | 0.562 | 1.320 | NA |
| 1560 | 0.488 | 0.966 | NA |
| 1561 | 1.015 | 2.453 | NA |
| 1562 | 0.936 | 2.166 | NA |
| 1563 | 0.877 | 2.175 | NA |
| 1564 | 0.701 | 2.715 | NA |
| 1565 | 1.161 | 1.957 | NA |
| 1566 | 0.612 | 2.457 | NA |
| 1567 | 1.269 | 4.068 | NA |
| 1568 | 0.154 | 2.000 | NA |
| 1569 | 0.029 | 1.365 | NA |
| 1570 | -0.252 | 2.037 | NA |
| 1571 | -0.491 | 1.495 | NA |
| 1572 | -0.750 | -1.380 | NA |
| 1573 | 0.028 | 0.987 | NA |
| 1574 | -1.294 | -1.714 | NA |
| 1575 | -0.874 | -0.275 | NA |
| 1576 | -0.723 | -0.138 | NA |
| 1577 | -0.113 | 2.164 | NA |
| 1578 | -0.157 | 1.849 | NA |
| 1579 | -0.910 | -1.359 | NA |
| 1580 | -0.575 | 0.423 | NA |
| 1581 | -0.046 | 0.456 | NA |
| 1582 | -0.423 | 0.436 | NA |
| 1583 | -0.205 | 0.461 | NA |
| 1584 | -0.010 | 1.107 | NA |
| 1585 | -0.187 | 0.099 | NA |
| 1586 | -0.156 | -0.153 | NA |
| 1587 | -0.599 | -0.647 | NA |
| 1588 | -0.102 | 0.637 | NA |
| 1589 | -0.705 | -1.576 | NA |
| 1590 | 0.075 | 0.605 | NA |
| 1591 | -0.106 | 0.269 | NA |
| 1592 | -0.202 | 0.706 | NA |
| 1593 | -1.284 | -0.978 | NA |
| 1594 | -0.685 | 0.444 | NA |
| 1595 | -0.905 | -0.661 | NA |
| 1596 | -0.174 | 1.187 | NA |
| 1597 | -0.337 | 1.079 | NA |
| 1598 | 0.030 | 1.267 | NA |
| 1599 | 0.253 | 2.145 | NA |
| 1600 | -0.328 | 0.047 | NA |
| 1601 | 0.131 | 1.653 | NA |
| 1602 | 0.414 | 2.626 | NA |
| 1603 | 0.137 | 1.187 | NA |
| 1604 | 0.281 | 1.378 | NA |
| 1605 | -0.825 | -1.285 | NA |
| 1606 | -0.545 | -1.014 | NA |
| 1607 | -1.154 | -1.592 | NA |
| 1608 | -0.565 | -0.698 | NA |
| 1609 | -1.179 | -0.979 | NA |
| 1610 | -0.318 | 0.849 | NA |
| 1611 | -0.956 | -0.939 | NA |
| 1612 | 0.340 | 2.747 | NA |
| 1613 | -1.854 | -4.031 | NA |
| 1614 | -0.649 | -0.989 | NA |
| 1615 | -0.744 | -1.035 | NA |
| 1616 | -1.344 | -2.019 | NA |
| 1617 | -0.637 | -1.254 | NA |
| 1618 | -0.284 | 0.881 | NA |
| 1619 | 0.077 | 1.100 | NA |
| 1620 | -0.354 | 0.965 | NA |
| 1621 | -0.218 | 0.490 | NA |
| 1622 | -0.289 | 0.360 | NA |
| 1623 | -0.231 | 0.635 | NA |
| 1624 | 0.461 | 2.347 | NA |
| 1625 | 0.079 | 1.298 | NA |
| 1626 | -0.592 | -0.218 | NA |
| 1627 | -0.716 | -1.588 | NA |
| 1628 | -1.642 | -2.514 | NA |
| 1629 | -1.326 | -1.135 | NA |
| 1630 | -1.435 | -2.424 | NA |
| 1631 | -1.604 | -2.746 | NA |
| 1632 | -1.128 | -2.350 | NA |
| 1633 | -0.421 | -0.446 | NA |
| 1634 | -0.467 | -0.366 | NA |
| 1635 | -1.661 | -2.911 | NA |
| 1636 | 0.133 | 1.238 | NA |
| 1637 | -0.536 | -0.400 | NA |
| 1638 | -1.724 | -2.776 | NA |
| 1639 | -1.188 | -0.763 | NA |
| 1640 | -0.810 | -0.413 | NA |
| 1641 | -0.238 | 1.553 | NA |
| 1642 | -0.008 | 1.741 | NA |
| 1643 | 0.091 | 0.399 | NA |
| 1644 | -0.196 | 0.120 | NA |
| 1645 | -0.105 | 0.194 | NA |
| 1646 | 0.454 | 2.230 | NA |
| 1647 | 0.555 | 0.745 | NA |
| 1648 | -0.369 | -1.327 | NA |
| 1649 | -1.159 | -0.594 | NA |
| 1650 | -0.095 | 0.659 | NA |
| 1651 | 0.647 | 1.499 | NA |
| 1652 | 0.057 | 0.291 | NA |
| 1653 | 0.406 | 0.514 | NA |
| 1654 | 0.153 | 1.132 | NA |
| 1655 | 0.219 | 1.263 | NA |
| 1656 | 0.165 | 0.599 | NA |
| 1657 | 0.389 | 0.962 | NA |
| 1658 | 0.769 | 1.462 | NA |
| 1659 | 1.014 | 2.562 | NA |
| 1660 | -0.340 | -0.072 | NA |
| 1661 | -1.356 | -3.556 | NA |
| 1662 | 0.146 | 1.186 | NA |
| 1663 | 0.426 | 1.327 | NA |
| 1664 | 0.612 | 1.161 | NA |
| 1665 | 1.148 | 1.957 | NA |
| 1666 | 0.785 | 1.956 | NA |
| 1667 | 0.883 | 2.061 | NA |
| 1668 | 0.480 | 0.883 | NA |
| 1669 | 0.849 | 2.340 | NA |
| 1670 | 0.951 | 3.035 | NA |
| 1671 | 0.480 | 1.407 | NA |
| 1672 | 0.505 | 0.912 | NA |
| 1673 | 0.527 | 1.566 | NA |
| 1674 | 0.118 | 1.256 | NA |
| 1675 | -2.093 | -0.883 | NA |
| 1676 | -1.373 | -0.859 | NA |
| 1677 | -0.477 | 0.314 | NA |
| 1678 | -0.023 | 1.186 | NA |
| 1679 | 0.177 | 2.086 | NA |
| 1680 | -0.232 | -0.766 | NA |
| 1681 | -0.091 | 3.087 | NA |
| 1682 | -0.058 | 0.355 | NA |
| 1683 | -1.792 | -3.795 | NA |
| 1684 | 0.596 | 1.894 | NA |
| 1685 | -0.265 | 0.707 | NA |
| 1686 | 0.347 | 2.298 | NA |
| 1687 | -1.311 | -2.284 | NA |
| 1688 | -0.101 | 0.703 | NA |
| 1689 | -0.548 | -1.074 | NA |
| 1690 | -1.179 | -1.356 | NA |
| 1691 | 0.136 | 0.414 | NA |
| 1692 | -0.026 | 0.845 | NA |
| 1693 | -0.583 | -2.304 | NA |
| 1694 | -0.151 | -0.304 | NA |
| 1695 | -0.352 | 0.001 | NA |
| 1696 | -0.005 | 1.356 | NA |
| 1697 | -0.954 | 0.088 | NA |
| 1698 | -0.504 | 0.549 | NA |
| 1699 | -0.369 | 0.863 | NA |
| 1700 | 0.172 | 1.796 | NA |
| 1701 | 0.088 | -0.932 | NA |
| 1702 | -0.290 | -0.431 | NA |
| 1703 | -0.542 | -1.502 | NA |
| 1704 | 0.435 | 1.036 | NA |
| 1705 | 0.605 | 0.952 | NA |
| 1706 | 0.926 | 1.063 | NA |
| 1707 | 1.399 | 2.385 | NA |
| 1708 | 1.189 | 1.597 | NA |
| 1709 | 0.964 | 1.383 | NA |
| 1710 | -0.037 | 0.947 | NA |
| 1711 | 0.480 | 2.267 | NA |
| 1712 | -0.347 | -0.112 | NA |
| 1713 | -0.368 | -0.374 | NA |
| 1714 | -0.714 | -0.865 | NA |
| 1715 | -0.276 | 0.045 | NA |
| 1716 | -0.212 | 0.273 | NA |
| 1717 | -0.068 | 0.142 | NA |
| 1718 | 0.119 | -0.215 | NA |
| 1719 | 0.567 | 1.147 | NA |
| 1720 | 0.116 | -0.138 | NA |
| 1721 | 0.151 | -0.424 | NA |
| 1722 | -0.579 | -0.605 | NA |
| 1723 | 0.899 | 1.997 | NA |
| 1724 | 0.700 | 1.242 | NA |
| 1725 | 0.458 | 0.745 | NA |
| 1726 | 0.872 | 2.149 | NA |
| 1727 | 1.279 | 2.541 | NA |
| 1728 | 0.832 | 2.055 | NA |
| 1729 | 0.115 | 0.195 | NA |
| 1730 | 0.088 | 0.909 | NA |
| 1731 | -0.912 | -1.098 | NA |
| 1732 | -0.361 | -0.830 | NA |
| 1733 | -0.172 | -0.891 | NA |
| 1734 | -1.953 | -4.958 | NA |
| 1735 | -0.161 | -0.747 | NA |
| 1736 | 0.103 | -0.149 | NA |
| 1737 | -0.177 | -0.561 | NA |
| 1738 | -1.330 | -4.380 | NA |
| 1739 | -0.482 | -1.686 | NA |
| 1740 | 0.072 | 0.289 | NA |
| 1741 | -0.014 | 0.248 | NA |
| 1742 | -0.228 | -0.286 | NA |
| 1743 | 0.408 | 1.496 | NA |
| 1744 | -0.452 | -1.191 | NA |
| 1745 | 0.185 | 1.171 | NA |
| 1746 | 0.169 | 0.831 | NA |
| 1747 | 0.021 | 0.086 | NA |
| 1748 | -0.331 | -0.457 | NA |
| 1749 | -0.048 | -0.471 | NA |
| 1750 | -0.442 | -1.181 | NA |
| 1751 | -0.069 | 0.660 | NA |
| 1752 | 0.484 | 1.582 | NA |
| 1753 | -0.430 | -0.403 | NA |
| 1754 | 0.380 | 1.415 | NA |
| 1755 | -0.830 | -0.180 | NA |
| 1756 | -0.180 | 0.327 | NA |
| 1757 | 0.050 | 2.107 | NA |
| 1758 | 0.012 | 1.771 | NA |
| 1759 | -0.598 | -1.858 | NA |
| 1760 | 0.008 | 1.807 | NA |
| 1761 | 0.148 | 1.136 | NA |
| 1762 | -1.174 | 1.511 | NA |
| 1763 | -0.197 | 2.290 | NA |
| 1764 | -0.321 | 0.985 | NA |
| 1765 | -0.238 | 0.719 | NA |
| 1766 | 0.578 | 2.133 | NA |
| 1767 | 0.819 | 3.057 | NA |
| 1768 | 0.790 | 2.551 | NA |
| 1769 | 0.611 | 2.191 | NA |
| 1770 | 0.189 | 1.573 | NA |
| 1771 | 0.870 | 2.414 | NA |
| 1772 | 0.987 | 1.956 | NA |
| 1773 | 0.834 | 1.618 | NA |
| 1774 | 0.845 | 2.193 | NA |
| 1775 | 0.901 | 1.632 | NA |
| 1776 | 0.176 | 1.528 | NA |
| 1777 | 0.377 | 0.537 | NA |
| 1778 | -0.273 | 1.537 | NA |
| 1779 | -0.166 | -0.639 | NA |
| 1780 | -0.497 | -0.617 | NA |
| 1781 | -0.822 | -1.804 | NA |
| 1782 | 0.240 | 0.663 | NA |
| 1783 | 0.385 | 0.510 | NA |
| 1784 | 0.993 | 2.415 | NA |
| 1785 | 1.410 | 3.503 | NA |
| 1786 | 1.478 | 3.523 | NA |
| 1787 | 1.614 | 2.889 | NA |
| 1788 | 1.859 | 4.384 | NA |
| 1789 | 1.004 | 1.807 | NA |
| 1790 | 0.728 | 0.463 | NA |
| 1791 | 0.135 | -0.253 | NA |
| 1792 | 0.124 | -0.328 | NA |
| 1793 | -0.163 | -0.588 | NA |
| 1794 | -0.764 | -1.761 | NA |
| 1795 | -0.777 | -1.999 | NA |
| 1796 | -0.402 | -1.448 | NA |
| 1797 | -0.167 | -1.001 | NA |
| 1798 | -0.569 | -1.953 | NA |
| 1799 | -0.006 | -0.356 | NA |
| 1800 | -0.172 | -0.935 | NA |
| 1801 | -0.811 | -2.212 | NA |
| 1802 | -0.412 | -1.827 | NA |
| 1803 | -0.274 | -0.718 | NA |
| 1804 | 0.362 | -0.138 | NA |
| 1805 | 0.608 | -0.570 | NA |
| 1806 | -0.249 | -1.752 | NA |
| 1807 | 0.318 | -0.658 | NA |
| 1808 | 0.588 | 0.525 | NA |
| 1809 | 0.934 | 0.192 | NA |
| 1810 | 0.202 | 0.570 | NA |
| 1811 | 0.293 | -0.015 | NA |
| 1812 | 0.077 | -0.653 | NA |
| 1813 | 0.310 | 0.388 | NA |
| 1814 | 0.692 | 1.397 | NA |
| 1815 | 1.080 | 1.506 | NA |
| 1816 | 0.724 | 1.247 | NA |
| 1817 | -0.132 | -0.988 | NA |
| 1818 | 0.140 | -0.110 | NA |
| 1819 | 0.452 | 0.714 | NA |
| 1820 | 0.032 | -0.487 | NA |
| 1821 | 0.327 | -1.198 | NA |
| 1822 | 0.404 | -0.603 | NA |
| 1823 | 0.577 | -0.024 | NA |
| 1824 | -0.143 | -2.344 | NA |
| 1825 | 0.409 | -0.910 | NA |
| 1826 | 0.709 | 0.844 | NA |
| 1827 | 0.661 | 0.619 | NA |
| 1828 | -0.350 | -1.761 | NA |
| 1829 | 0.466 | 0.770 | NA |
| 1830 | 0.515 | 1.138 | NA |
| 1831 | 0.962 | 0.866 | NA |
| 1832 | 1.213 | 2.446 | NA |
| 1833 | 1.292 | 2.249 | NA |
| 1834 | 0.110 | -0.530 | NA |
| 1835 | 1.401 | 2.811 | NA |
| 1836 | 0.576 | 1.406 | NA |
| 1837 | 1.405 | 3.300 | NA |
| 1838 | 1.331 | 1.855 | NA |
| 1839 | 0.713 | 0.668 | NA |
| 1840 | 0.848 | 1.298 | NA |
| 1841 | 0.384 | -0.628 | NA |
| 1842 | 0.386 | 0.093 | NA |
| 1843 | 0.087 | -0.449 | NA |
| 1844 | -0.042 | -0.598 | NA |
| 1845 | 0.272 | 0.413 | 0.565 |
| 1846 | 0.593 | 0.918 | 1.544 |
| 1847 | 0.159 | 0.477 | 0.802 |
| 1848 | 0.134 | 0.937 | 1.315 |
| 1849 | 0.249 | 1.611 | 2.213 |
| 1850 | -0.534 | -1.262 | -0.842 |
| 1851 | 0.286 | 1.511 | 1.936 |
| 1852 | -0.074 | 0.581 | 0.750 |
| 1853 | 0.442 | 1.446 | 1.938 |
| 1854 | 0.145 | 0.877 | 1.218 |
| 1855 | 1.018 | 2.547 | 3.125 |
| 1856 | 0.702 | 1.865 | 2.162 |
| 1857 | 0.290 | 1.501 | 1.102 |
| 1858 | -0.165 | 0.302 | -0.344 |
| 1859 | -0.509 | -1.036 | -1.450 |
| 1860 | -0.134 | 0.048 | -0.227 |
| 1861 | -0.930 | -0.760 | -0.746 |
| 1862 | -1.295 | -2.255 | -1.994 |
| 1863 | -0.576 | -0.667 | -0.675 |
| 1864 | -0.557 | -0.636 | -0.281 |
| 1865 | -0.585 | -0.071 | -0.079 |
| 1866 | -0.645 | -0.446 | -0.684 |
| 1867 | -0.885 | -2.177 | -2.753 |
| 1868 | -0.589 | -0.541 | -1.134 |
| 1869 | -0.683 | -0.994 | -1.182 |
| 1870 | -0.267 | 0.390 | 0.473 |
| 1871 | -0.110 | 0.153 | 0.387 |
| 1872 | -0.062 | 0.367 | 0.437 |
| 1873 | 0.024 | 0.629 | 1.062 |
| 1874 | -0.508 | -2.316 | -3.492 |
| 1875 | -0.421 | 0.251 | 0.373 |
| 1876 | -0.187 | 0.033 | 0.197 |
| 1877 | -0.039 | -1.072 | -1.149 |
| 1878 | -0.991 | -4.175 | -5.735 |
| 1879 | -1.446 | -1.856 | -2.866 |
| 1880 | -0.682 | -0.811 | -1.381 |
| 1881 | -0.699 | -2.055 | -2.517 |
| 1882 | -1.752 | -5.012 | -6.617 |
| 1883 | -0.375 | -1.511 | -2.297 |
| 1884 | 0.054 | -0.335 | -1.168 |
| 1885 | -0.059 | -0.168 | -0.587 |
| 1886 | 0.098 | 0.072 | -0.394 |
| 1887 | -0.554 | -2.164 | -2.959 |
| 1888 | 0.178 | 0.600 | 0.641 |
| 1889 | 0.087 | -0.027 | -0.135 |
| 1890 | 0.088 | -0.252 | -0.531 |
| 1891 | 0.039 | -0.448 | -0.452 |
| 1892 | 0.045 | -1.875 | -2.454 |
| 1893 | 0.568 | -0.733 | -1.243 |
| 1894 | 0.250 | -0.295 | -1.015 |
| 1895 | -0.026 | -0.712 | -1.130 |
| 1896 | -0.030 | -0.207 | -0.930 |
| 1897 | -0.455 | -1.717 | -2.563 |
| 1898 | 0.164 | -0.018 | -0.768 |
| 1899 | -0.843 | -0.449 | -0.507 |
| 1900 | -0.296 | 0.447 | 0.479 |
| 1901 | 0.005 | 1.157 | 1.862 |
| 1902 | 0.369 | 0.779 | 1.066 |
| 1903 | -0.011 | 0.670 | 0.856 |
| 1904 | -0.271 | 0.182 | -0.535 |
| 1905 | -0.670 | -1.335 | -2.668 |
| 1906 | -0.212 | 0.435 | -0.061 |
| 1907 | 0.011 | 0.603 | -0.127 |
| 1908 | 0.269 | 0.686 | 0.224 |
| 1909 | -0.249 | -0.413 | -0.793 |
| 1910 | 0.020 | -0.176 | -0.731 |
| 1911 | 0.282 | 0.524 | -0.249 |
| 1912 | 0.480 | 0.991 | 1.041 |
| 1913 | -0.850 | -0.621 | -0.478 |
| 1914 | -0.780 | -0.621 | -0.310 |
| 1915 | -0.830 | -0.655 | -0.437 |
| 1916 | -0.505 | -0.463 | -0.723 |
| 1917 | -0.587 | -0.564 | -0.502 |
| 1918 | -0.241 | -0.641 | -0.748 |
| 1919 | 0.022 | -0.640 | -0.643 |
| 1920 | 0.688 | 0.805 | 0.254 |
| 1921 | 0.902 | 1.476 | 1.381 |
| 1922 | 0.616 | 1.480 | 2.057 |
| 1923 | 0.636 | 1.597 | 1.362 |
| 1924 | 0.300 | 1.441 | 1.239 |
| 1925 | 0.708 | 1.362 | 1.228 |
| 1926 | 0.038 | -0.799 | -0.948 |
| 1927 | 0.515 | 1.593 | 1.143 |
| 1928 | 0.634 | 1.658 | 1.308 |
| 1929 | -0.092 | -0.265 | 0.601 |
| 1930 | -0.222 | 0.966 | 2.272 |
| 1931 | -0.435 | -0.871 | -0.903 |
| 1932 | 0.127 | 0.203 | 0.482 |
| 1933 | -0.850 | -2.193 | -1.967 |
| 1934 | -0.861 | -1.244 | -1.288 |
| 1935 | -0.185 | -0.591 | -0.190 |
| 1936 | 0.295 | 0.411 | 1.256 |
| 1937 | 0.428 | -0.824 | -0.987 |
| 1938 | 0.517 | 0.531 | 0.811 |
| 1939 | 0.457 | 0.655 | 1.253 |
| 1940 | 0.345 | 0.135 | 0.906 |
| 1941 | 0.814 | 1.844 | 2.719 |
| 1942 | 0.719 | 1.748 | 2.315 |
| 1943 | 0.922 | 2.386 | 2.834 |
| 1944 | 0.922 | 1.912 | 2.942 |
| 1945 | -0.446 | -2.723 | -4.616 |
| 1946 | 0.772 | 1.405 | 1.817 |
| 1947 | 0.984 | 1.489 | 2.108 |
| 1948 | 0.524 | 0.788 | 0.986 |
| 1949 | 0.089 | -0.316 | -0.184 |
| 1950 | -0.851 | -1.898 | -1.981 |
| 1951 | 0.081 | 0.149 | 0.314 |
| 1952 | 0.502 | -0.163 | -0.395 |
| 1953 | 0.502 | 0.179 | -1.035 |
| 1954 | 0.756 | 1.401 | 0.993 |
| 1955 | 0.831 | 1.601 | 1.634 |
| 1956 | 0.515 | 1.111 | 1.053 |
| 1957 | -0.899 | -1.363 | -1.513 |
| 1958 | -0.603 | -0.365 | 0.262 |
| 1959 | -0.563 | -0.663 | -0.291 |
| 1960 | 0.824 | 1.389 | 2.398 |
| 1961 | 0.479 | 0.564 | 1.215 |
| 1962 | 0.901 | 1.617 | 2.175 |
| 1963 | 0.974 | 3.329 | 4.401 |
| 1964 | 0.209 | 1.892 | 2.511 |
| 1965 | 0.618 | 2.137 | 2.463 |
| 1966 | 0.832 | 1.231 | -0.011 |
| 1967 | 0.364 | 0.775 | 0.773 |
| 1968 | 1.236 | 1.777 | 1.520 |
| 1969 | 1.761 | 1.894 | 2.840 |
| 1970 | 0.077 | 0.779 | 1.575 |
| 1971 | -1.719 | -0.851 | 0.321 |
| 1972 | -0.987 | -0.612 | 0.478 |
| 1973 | -0.354 | 0.062 | 1.301 |
| 1974 | -0.111 | -0.039 | 0.714 |
| 1975 | -0.678 | 0.096 | 1.483 |
| 1976 | -1.034 | 0.387 | 1.821 |
| 1977 | -0.835 | -1.029 | -0.430 |
| 1978 | 0.189 | 0.939 | 2.673 |
| 1979 | -0.885 | -0.610 | 0.165 |
| 1980 | -1.643 | -2.382 | -1.394 |
| 1981 | -1.454 | -3.700 | -4.100 |
| 1982 | -0.305 | -1.412 | -1.667 |
| 1983 | -1.073 | -3.682 | -4.714 |
| 1984 | -0.545 | -2.667 | -3.220 |
| 1985 | -0.255 | NA | NA |
| 1986 | -0.040 | NA | NA |
| 1987 | -0.070 | NA | NA |
| 1988 | 0.366 | NA | NA |
| 1989 | 0.533 | NA | NA |
| 1990 | 0.673 | NA | NA |
| 1991 | 1.286 | NA | NA |
| 1992 | 1.182 | NA | NA |
| 1993 | -0.089 | NA | NA |
| 1994 | 0.004 | NA | NA |
| 1995 | -0.885 | NA | NA |
| 1996 | 0.801 | NA | NA |
| 1997 | 0.793 | NA | NA |
| 1998 | -1.458 | NA | NA |
| 1999 | -1.265 | NA | NA |
| 2000 | -1.005 | NA | NA |
| 2001 | -0.577 | NA | NA |
| 2002 | -0.311 | NA | NA |
| 2003 | -1.438 | NA | NA |
| 2004 | -0.594 | NA | NA |
| 2005 | -0.570 | NA | NA |
| 2006 | -0.555 | NA | NA |
| 2007 | -0.588 | NA | NA |
| 2008 | -1.368 | NA | NA |
| 2009 | -0.881 | NA | NA |
| 2010 | -0.157 | NA | NA |
| 2011 | 0.241 | NA | NA |
| 2012 | 0.094 | NA | NA |
| 2013 | 0.965 | NA | NA |
